# Detection of Victoria lineage influenza B viruses with K162 and N163 deletions in the hemagglutinin gene, South Africa, 2018

**DOI:** 10.1002/hsr2.367

**Published:** 2021-09-17

**Authors:** Orienka Hellferscee, Florette Treurnicht, Lucinda Gaelejwe, Alexandra Moerdyk, Gary Reubenson, Meredith McMorrow, Stefano Tempia, Johanna McAnerney, Sibongile Walaza, Nicole Wolter, Anne von Gottberg, Cheryl Cohen

**Affiliations:** ^1^ Centre for Respiratory Diseases and Meningitis National Institute for Communicable Diseases of the National Health Laboratory Service Johannesburg South Africa; ^2^ Department of Medical Virology, School of Pathology, Faculty of Health Sciences University of the Witwatersrand Johannesburg South Africa; ^3^ Department of Paediatrics & Child Health, Faculty of Health Sciences University of the Witwatersrand Johannesburg South Africa; ^4^ Influenza Division Centers for Disease Control and Prevention Atlanta Georgia USA; ^5^ Influenza Program Centers for Disease Control and Prevention Pretoria South Africa; ^6^ MassGenics Duluth Georgia USA; ^7^ School of Public Health, Faculty of Health Sciences University of the Witwatersrand Johannesburg South Africa; ^8^ School of Pathology, Faculty of Health Sciences University of the Witwatersrand Johannesburg South Africa

**Keywords:** deletions, influenza B, South Africa, Victoria lineage

## Abstract

**Background:**

A group of Victoria lineage influenza B viruses with a two amino acid deletion in the hemagglutinin (HA) at residues K162 and N163, was detected during the 2016 to 2017 Northern Hemisphere influenza season and continues to spread geographically. We describe the first identification of viruses with these deletions from South Africa in 2018.

**Methods:**

Nasopharyngeal samples were obtained from the syndromic surveillance programs. Real‐time reverse transcription‐polymerase chain reaction was used for virus detection and lineage determination. Influenza genetic characterization was done using next‐generation sequencing on the MiSeq platform. The duration of virus circulation was determined using thresholds calculated using the Moving Epidemic Method; duration was used as an indicator of disease transmissibility and impact.

**Results:**

In 2018, 42% (426/1015) of influenza‐positive specimens were influenza B viruses. Of 426 influenza B‐positive samples, 376 (88%) had the lineage determined of which 75% (283/376) were Victoria lineage. The transmissibility of the 2018 South African influenza season was high for a few weeks, although the severity remained moderate through most of the season. The sequenced 2018 South African Victoria lineage influenza B viruses clustered in sub‐clade V1A.1 with the 162‐163 deletions.

**Conclusions:**

We report the first detection of the 162‐163 deletion variant of influenza B/Victoria viruses from South Africa in 2018, and suggest that this deletion variant replaced the previous circulating influenza B/Victoria viruses. These deletions putatively affect the antigenic properties of the viruses because they border an immune‐dominant region at the tip of the HA. Therefore, close monitoring of these newly emerging viruses is essential.

## 1 BACKGROUND

Seasonal influenza viruses cause up to 5 million cases of severe illness and approximately 500 000 deaths globally each year.^1^ The burden of influenza disease varies from year to year, depending on the circulating strain(s), vaccination coverage, and vaccine‐virus circulating‐virus match (although this might be less relevant in settings where the vaccine coverage is low). Influenza B virus, in general, affects younger individuals than influenza A.^2^

Influenza B viruses were first described in 1940 and since the 1980s, two lineages of influenza B viruses have circulated with distinct antigenicity and transmission dynamics.^2^, ^3^ Historically there has been less focus on influenza B because earlier studies suggested that influenza B caused less disease than influenza A during annual influenza epidemics and it does not pose a pandemic threat.^2^ The epidemiology of influenza B Victoria and Yamagata lineage viruses differ, including the younger average age of persons infected with Victoria lineage viruses, but no differences by clinical presentation.^4^, ^5^, ^6^

Typically, the South African influenza season starts with influenza A activity and ends with an influenza B “tail”.^7^ The mean onset of the South African influenza season is week 23.^8^ South Africa used the trivalent influenza vaccine in 2018, which contains two influenza A viruses subtypes, A(H3N2) and A(H1N1)pdm09, and one influenza B virus lineage (Yamagata), based on the Southern hemisphere recommendations from the World Health Organization.^9^ Since 2020, a quadrivalent vaccine, which contains both of the influenza B lineages is also available for use in South Africa.

Both influenza A and B are prone to antigenic drift, although influenza B viruses evolve more slowly than influenza A. Influenza B viruses are known for insertions and deletions in the hemagglutinin and neuraminidase genes during evolution.^10^, ^11^, ^12^ An influenza B Victoria virus strain with two amino acid deletions in the hemagglutinin gene at residues K162 and N163, designated as V1A.1, was detected during the 2016 to 2017 Northern Hemisphere influenza season. Since these Victoria lineage influenza B viruses with deletions in the HA were not detected in South Africa previously, we aimed to determine whether viruses with amino acid deletions K162 and N163 in the hemagglutinin gene, were circulating in South Africa in 2018.

## 2 METHODS

### 2.1 Surveillance programmes

For this study, we used data from two main influenza surveillance programmes in South Africa, which are coordinated by the Centre for Respiratory Diseases and Meningitis (CRDM) at the National Institute for Communicable Diseases (NICD) of the National Health Laboratory Service (NHLS), Johannesburg, South Africa. These programmes include pneumonia surveillance among hospitalized patients and influenza‐like illness surveillance in outpatients at private general practitioners, the Viral Watch programme (VW) (Table 1).

**TABLE 1:** Description of influenza surveillance programmes in South Africa, 2018

Program	Viral watch	Syndromic surveillance for pneumonia
Start year	1984	2009
Provinces^a^	EC, FS, GP, LP, MP, NC, NW, WC	GP, KZ, MP, NW, WC
Type of site	General practitioners (mainly private)	Public hospitals
Case definition	An acute respiratory illness with a fever (≥38°C) or history of fever, cough, and symptom onset within the last 10 days	Infants (aged 2 days to <3 month): Any patient with a diagnosis of suspected sepsis or physician‐diagnosed acute lower respiratory tract infection (LRTI), irrespective of signs and symptoms Children (aged 3 months to <5 years): Any patient with physician‐diagnosed acute LRTI or pleural effusion Patients aged ≥5 years: An acute or chronic LRTI with fever (≥38°C) or history of fever and cough among hospitalized patients, regardless of symptom onset

^a^ EC, Eastern Cape; FS, Free State; GP, Gauteng Province; KZ, KwaZulu‐Natal; LP, Limpopo Province; MP, Mpumalanga Province: NC, Northern Cape; NW, North West; WC, Western Cape.

### 2.2 Study procedures

The procedures for syndromic surveillance for pneumonia have been previously described.^8^, ^13^, ^14^, ^15^, ^16^ In brief, study staff completed case report forms and collected combined nasopharyngeal and oropharyngeal (NPOP) swabs for all enrolled inpatients. For the Viral Watch programme, general practitioners completed a case report form and collected NPOP swabs for all enrolled outpatients.^8^

### 2.3 Sample collection and real‐time RT‐PCR influenza virus detection

Combined NPOP swabs were placed in a universal transport medium (Copan, Murrieta, CA), stored at 4°C to 8°C, and transported within 72 hours to NICD for testing. All specimens were extracted using 200 μL and eluted in 100 μL on the MagNA Pure 96 instrument, Total Nucleic Acid Small Volume Kit (Roche, Basel, Switzerland). The extracts were tested for influenza A and B viruses and respiratory syncytial virus (RSV) using Fast Track Diagnostics (FTD) FLU/HRSV assay (FTD, Luxembourg) on the ABI 7500 Fast instrument, according to the manufacturer's instructions.

### 2.4 Real‐time RT‐PCR assays for influenza B lineage determination

Influenza B lineage typing, using the CDC real‐time one‐step RT‐PCR lineage typing kits obtained through the International Reagent Resource Program (https://www.influenzareagentresource.org), was performed for all influenza B positive samples (Ct value <40) as per manufacturer's instructions. Influenza B lineage results were assigned as “inconclusive” when no lineage could be established due to insufficient viral load (Ct value ≥35).

### 2.5 Influenza genetic characterization using next‐generation sequencing on the MiSeq platform

All influenza B/Victoria positive samples were extracted using 200 μL and eluted in 50 μL on the MagNA Pure 96 instrument using the Total Nucleic Acid Small Volume Kit. The genomic segments of influenza B viruses were amplified simultaneously. Briefly, nucleic acid extracts were treated with RNase‐free DNase I (Invitrogen, California, USA) to enrich RNA. The enriched RNA served as a template in the PCR reaction mixture which combined 20 μM of primers (B‐uniF1: GGG GGG AGC AGA AGC RG; B‐uniF2: GGG GGG AGC AGA AGC AC; B‐uniR1: CCG GGT TAT TAG TAG AAA CAA C; B‐uniR2: CCG GGT TAT TAG TAG WAA CAM G) with SuperScript III one‐step RT‐PCR system with Platinum *Taq* high‐fidelity DNA polymerase system (ThermoFisher, Massachusetts, USA).^17^, ^18^ PCR products were confirmed on a 1% agarose gel. PCR amplicons were enriched for viral templates by digestion of host genomic DNA using MspJI restriction enzyme (New England Biolabs, Massachusetts, USA).^19^ Specimens were then processed for next‐generation sequencing on the Illumina MiSeq instrument.

Illumina sequencing library was prepared from amplified PCR products using Nextera XT Sample Preparation kit (Illumina, California, USA). Samples were multiplexed and sequenced using Illumina Miseq v3 kit with 300‐bp paired‐end reads.

Reads were de‐multiplexed following the removal of tags and adaptors. Following importation in CLC Genomics Workbench version 11 (Qiagen, Netherlands), reads were paired, trimmed, and merged using the following parameters: Trim quality score limit = 0.01, Trim ambiguous nucleotides with a maximum number of ambiguities = 1. After trimming, reads shorter than 50 nucleotides were discarded. The resultant sequenced reads were analysed using a reference‐based mapping approach with the following parameters: mismatch cost = 10, insertion cost = 3, deletion cost = 3, and length fraction = 0.5, Similarity fraction = 0.95. The consensus sequence was extracted and exported for further analysis. Consensus were 100× in coverage and did not include date under Q30.

### 2.6 Phylogenetic analysis

The genetic diversity of full‐length NA and HA consensus sequences were determined by alignment with international reference strains from each clade downloaded (October 7, 2019) from Global Initiative on Sharing All Influenza Data (GISAID) (https://www.gisaid.org)—including South African 2016 influenza B Victoria strains for comparison and the reference virus for group V1A.1 viruses represented by B/Colorado/06/2017. Multiple sequence alignments were generated in the MAFFT version 7 (Multiple Alignment using Fast Fourier Transform) multiple sequence alignment program^20^ and analyzed in the BioEdit Sequence Alignment Editor Software version 7.0.5.3.^21^

Maximum‐likelihood trees were constructed using the Tamura‐Nei model with 1000 bootstraps using MEGA version 7.^22^ The 2018 sequences downloaded from GISAID (all geographical locations)^23^ were used to construct phylogenetic trees, but only sequences clustering together with South African viruses were kept in the trees for better visualization.

NA sequences generated were screened for the presence of known molecular markers of neuraminidase inhibitor (Oseltamivir) resistance that demonstrated clinical relevance in influenza B viruses (R150K, D197E).^24^, ^25^

### 2.7 Assessment of the transmissibility and impact of the 2018 influenza B season

We used the Moving Epidemic Method (MEM), a sequential analysis using the R language (http://CRAN.R-project.org/web/package=mem) to establish thresholds for influenza B transmissibility and impact (hereafter referred to as indicators) using influenza surveillance data during 2012 to 2017.^26^ We compared the established threshold to the weekly value of each indicator in 2018. The weekly proportion of samples testing positive for influenza B (irrespective of lineage) from the outpatient Viral Watch programme were used as the proxy indicator for transmissibility. A similar proportion among inpatients from the pneumonia surveillance programme was used as the proxy indicator for impact. This procedure was in line with the WHO guidelines on Pandemic Influenza Severity Assessment which is also recommended for use during annual seasonal influenza.^27^ MEM uses historical data to determine an epidemic threshold as well as moderate, high, and very high activity thresholds for the selected indicator(s). Epidemic and activity thresholds are usually calculated using data from 5 to 10 historical seasons. We used the 40th, 90th, and 97.5th percentiles of the MEM estimates as recommended by WHO to obtain the threshold value for moderate, high, and very high activity, respectively for each of the two indicators.^27^ The epidemic thresholds estimated by MEM were used to define the beginning and the end of the influenza season. Values below the epidemic threshold were considered pre‐ or post‐epidemic. The weekly value of each indicator for the 2018 influenza season was compared to the historical thresholds, rating each week as follows: below threshold (below epidemic threshold), low activity (between epidemic and moderate activity thresholds), moderate activity (between moderate and high activity thresholds), high activity (between high and very high activity thresholds) and very high activity (above very high activity threshold).

## 3 RESULTS

### 3.1 Description of the 2018 South African influenza season

**FIGURE 1:**
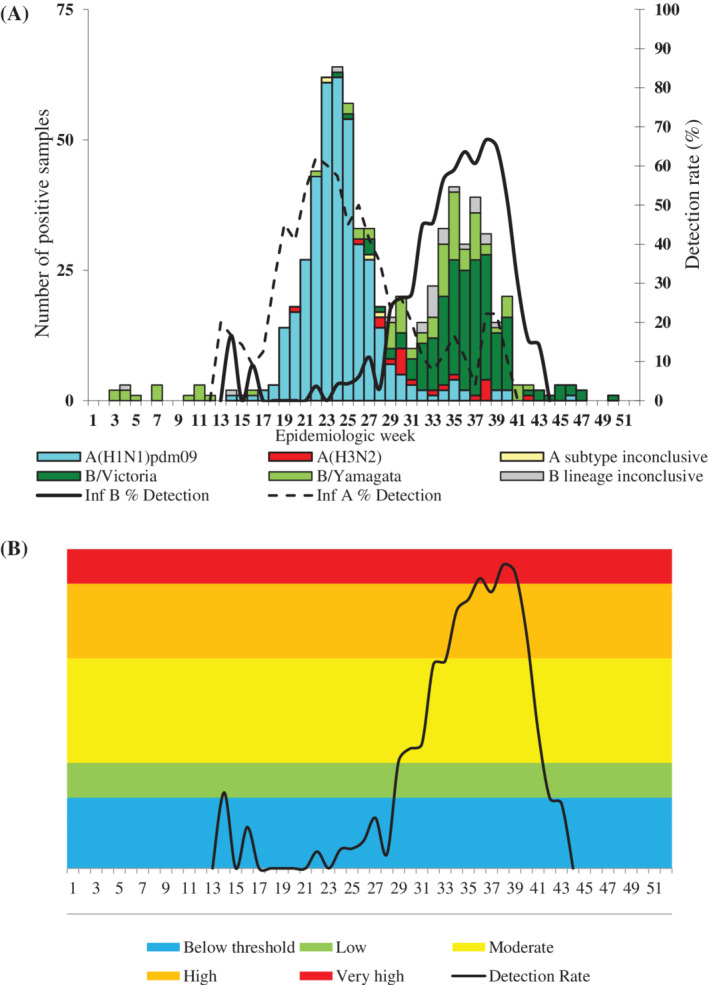
Influenza A and B positive samples by lineage and detection rate for 2018 A, and moving epidemic method (MEM) B, (thresholds based on 2012‐2017 data) for influenza B, Viral Watch surveillance programme

**FIGURE 2:**
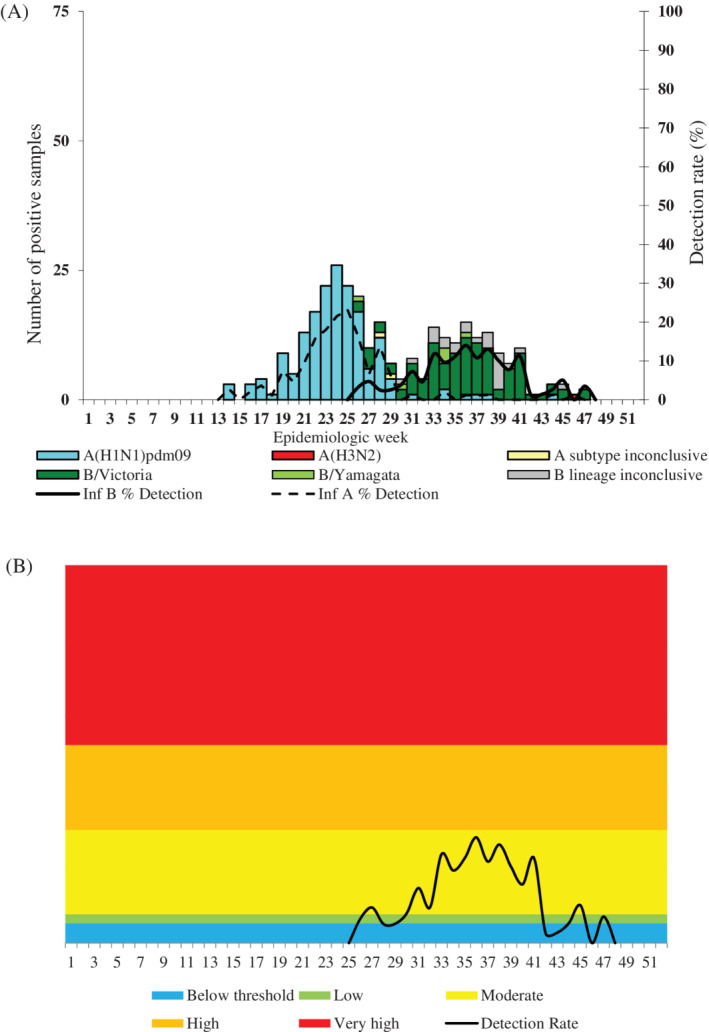
Influenza A and B detections by lineage and detection rate for 2018 A, and moving epidemic method (MEM) B, (thresholds based on 2012‐2017 data) for influenza B, pneumonia surveillance programme

In 2018, 17% (1015/6096) of samples tested positive for influenza viruses (Table 2). The 2018 influenza season was characterized by first wave of influenza A(H1N1)pdm09 followed by a second wave of influenza B (Figures 1A and 2A). Overall, 42% (426/1015) of influenza‐positive specimens tested positive for influenza B (41% for Viral Watch and 44% for pneumonia surveillance) (Table 2). Among influenza B‐positive samples for which lineage was determined, 75% (283/376) were Victoria lineage (Table 2). The South African influenza B season peaked in week 38, reaching very high transmissibility in weeks 36, 38, and 39 compared to historical thresholds (2012‐2017) (Figure 1B). The 2015 and 2017 influenza B tails were dominated by B Yamagata in contrast to 2016, where the whole influenza season was dominated by B Victoria (Figure S1A,B).

**TABLE 2:** Total number of influenza cases in syndromic influenza surveillance programmes, South Africa, 2018

Programme	Specimens tested	Influenza positive	Influenza B	B Victoria	B Yamagata	B inconclusive lineage
	N	N (%)	N (%)	N (%)	N (%)	N (%)
Viral Watch	1465	712 (49)	293 (41)	181 (62)	87 (30)	25 (8)
Pneumonia surveillance	4631	303 (7)	133 (44)	102 (77)	6 (5)	25 (19)
Total	6096	1015 (17)	426 (42)	283 (66)	93 (22)	50 (12)

## 4 GENETIC CHARACTERIZATION

### 4.1 Hemagglutinin gene analysis

Only 20% (56/283) of all B/Victoria positive samples tested with the genomic PCR produced visible bands on gel electrophoresis and were sequenced. Only 26/56 (46%) samples yielded full‐length hemagglutinin (HA). Unfortunately, the remaining 30 samples did not yield sequences sufficient to determine if the two deletions were present. All (26/26) B/Victoria specimens isolated from South Africa in 2018 clustered in sub‐clade V1A.1 (2 del) and contained both deletions at positions K162 and N163.

**FIGURE 3:**
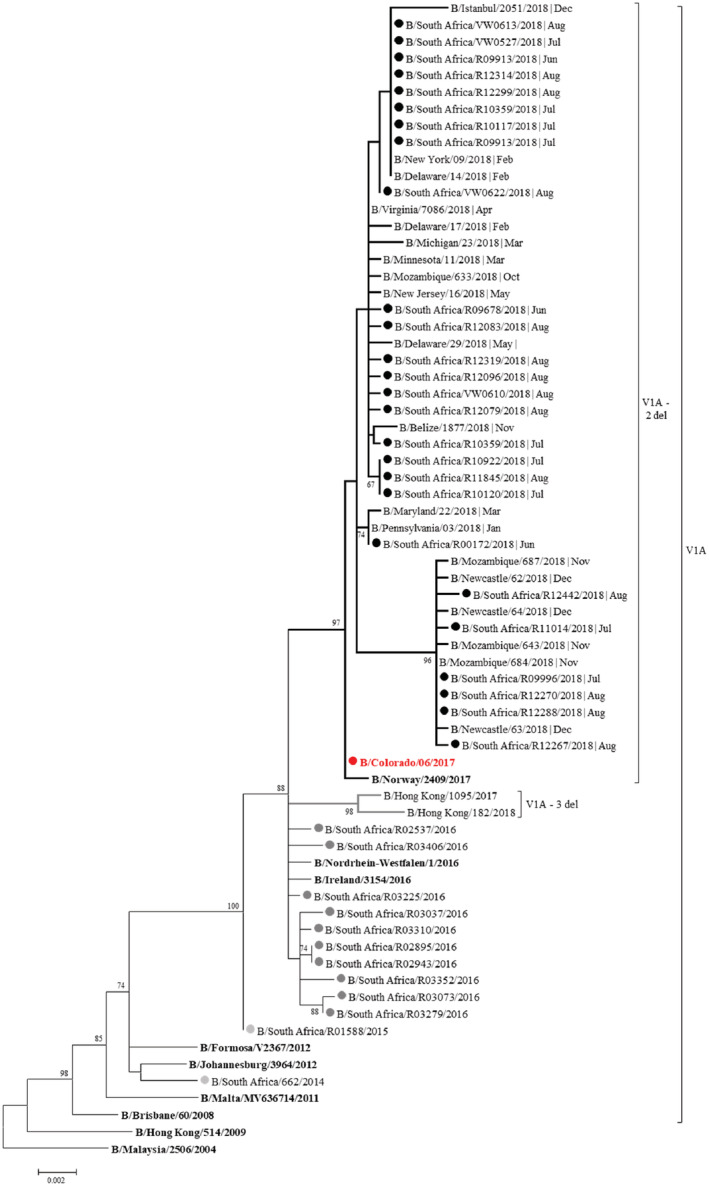
Maximum likelihood tree of the hemagglutinin (HA) gene of influenza B Victoria strains (1713 bp). Black dots = 2018 South African strains; dark gray dots = 2016 South African strains; light gray dots = 2014 to 2015 South African strains; red dot = 2018/2019 Northern hemisphere, and 2019 Southern hemisphere vaccine strain

Group V1A.1, represented by B/Colorado/06/2017, shares amino acid changes at HA residues I117V, N129G, V146I, I180V, R498K, and includes two amino acid deletions in the HA at residues K162 (K162del) and N163 (N163del) compared to B/Brisbane/60/2008 (Figure 3). None of the K162‐N163 deletions were seen in 2016 South African strains (n = 10) previously sequenced (B/Victoria did not circulate in South Africa in 2017) (Figure 3).

The majority of the sequences (19/26, 73%) were from samples submitted from the Western Cape and clustered together in a large clade within V1A.1 with viruses from the United States of America, Mozambique, and Europe isolate in 2018 (Figure 3). One VW sample (R00172/2018) from a Gauteng Province VW general practitioner clustered separately together with viruses from the United States isolated in January and March 2018.

Six of the 26 South African 2018 B/Victoria viruses formed a separate cluster with 96% support (Figure 3)—this was due to 7 point mutations that did not translate into amino acid changes (T372C, G837A, C984T, G1074A, T1299C, G1419A, and G1551A). Five of six samples isolated from patients in Gauteng clustered in this separate clade, compared to samples isolated in the Western Cape (R12442/2018, R12270/2018, R09996/2018, R11014/2018, and R12267/2018). Only one sample from the Western Cape clustered with the Gauteng cluster (R12288/2018). The South African viruses from Gauteng clustered with viruses from Mozambique and Australia (Newcastle, New South Wales) isolated in 2018.

## 5 NEURAMINIDASE GENE ANALYSIS

Of the 56 samples sequenced in this study, only 16 (29%) samples yielded full‐length neuraminidase (NA) sequences and all 16 clustered in sub‐clade V1A.1 (2 del). Unfortunately, the remaining 40 samples did not yield sequences sufficient to determine if they clustered with the V1A.1 subclade. All clusters identified with HA were reinforced by the NA analysis.

**FIGURE 4:**
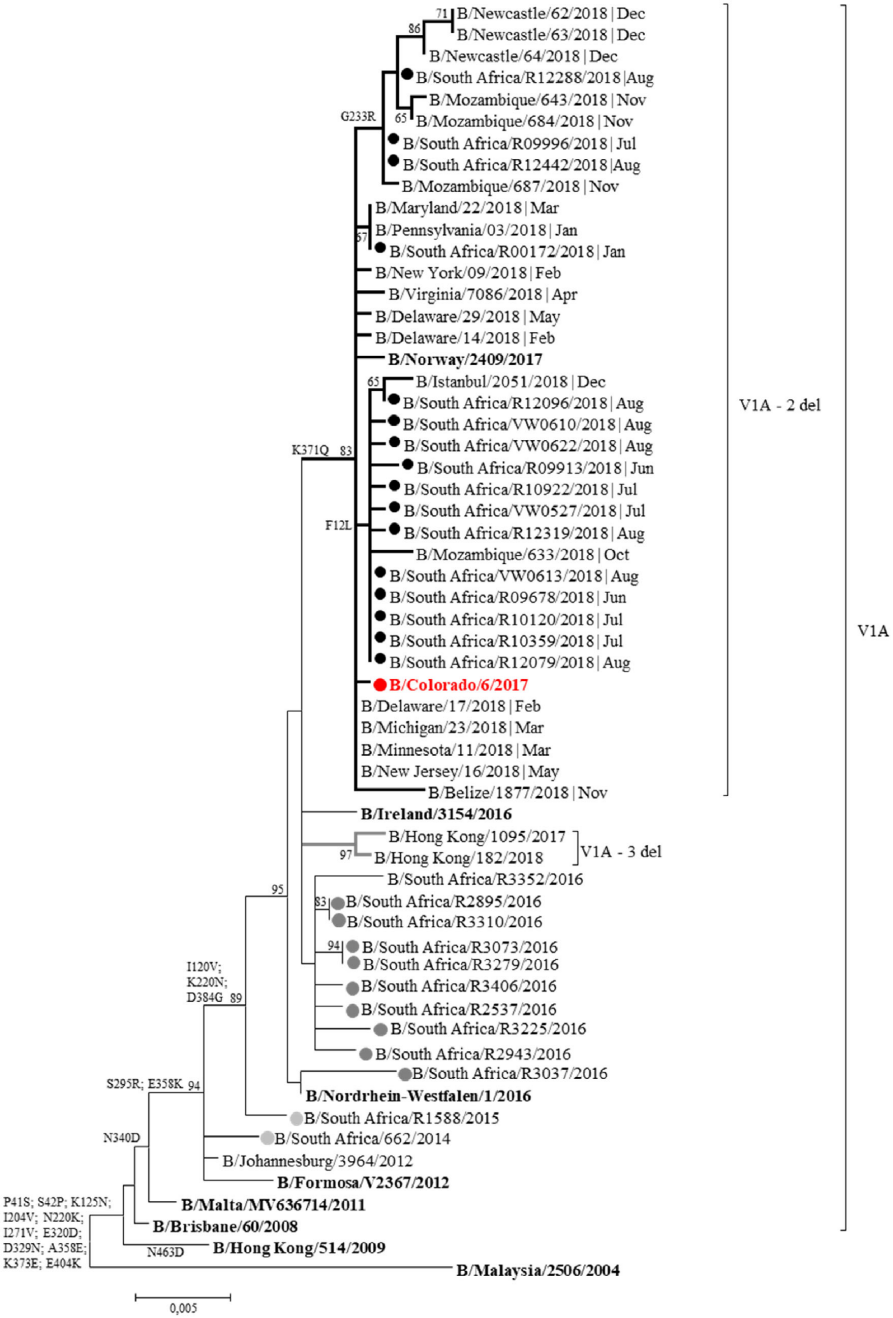
Maximum likelihood tree of the neuraminidase (NA) gene of influenza B Victoria trains (1401 bp). Black dots = 2018 South African strains; dark gray dots = 2016 South African strains; light gray dots = 2014 to 2015 South African strains; red dot = 2018/2019 Northern hemisphere and 2019 Southern hemisphere vaccine strain

Group V1A.1, represented by B/Colorado/06/2017, contains amino acid changes at NA residue K371Q (Figure 4). Like the HA analysis, the majority (75%, 12/16) of the 2018 South African influenza B Victoria viruses had mutation F12L, clustering with viruses from the United States, Mozambique, and Europe. Three South African viruses (19%) had mutation G233R, clustering with viruses from Mozambique and Australia (Newcastle, New South Wales) and one South African virus had mutation T211I, clustering with viruses from the United States.

All 2018 South African influenza B Victoria viruses with NA sequence data available (16/16) had an oseltamivir sensitive genotype (R150 and D197 in the NA gene).

## 6 CONCLUSIONS

In South Africa, the 2018 influenza season was characterized by the circulation of influenza B viruses toward the end of the influenza season. The Victoria lineage dominated and all the viruses sequenced had the K162 and N163 deletions in the HA gene. This strain has not been detected previously during Victoria lineage seasons in South Africa (2016). The circulating South African influenza B viruses were more transmissible in 2018 compared to previous years but not more severe.

In 2018 in South Africa, almost half of influenza‐positive specimens tested positive for influenza B and Victoria was the dominant lineage, the first time since 2016. Influenza B seasons with alternating years of lineage dominance was the regular pattern in previous seasons in the Southern hemisphere (Figure S1A,B), inter‐tropical belt, and Northern hemisphere countries during 2000 to 2014.^28^ All the 2018 South African Victoria lineage influenza B viruses sequenced clustered in sub‐clade V1A.1 containing the 162‐163 deletions. The emergence of these B Victoria lineage viruses with 2 amino acid deletions in the HA was first reported in the United States during the 2016 to 2017 season.^29^ Subsequently, it has been detected commonly in the United States and Europe.^30^ Since 2018, viruses with a three amino acid deletion in the HA (162‐164) belonging to subclades V1A.2 and V1A.3, which are antigenically distinct from V1A.1 viruses, predominated in most countries. However, in Madagascar, Mozambique, and many countries in Central and South America, similar to South Africa, viruses with the two amino acid deletion dominated.^31^

The South African 2018 Victoria lineage influenza B viruses formed two clusters, one in the Western Cape and one in Gauteng. The separation of these two clusters was due to 7 point mutations (T372C, G837A, C984T, G1074A, T1299C, G1419A, and G1551A), none of which was associated with an amino acid change. Influenza viruses evade immune detection through the development of mutations in surface proteins (antigenic drift).^32^ Influenza B viruses can acquire insertions and deletions in the HA and NA gene segments during evolution,^12^ and certain HA deletions might affect the antigenic properties of the viruses since they border an immune‐dominant region at the tip of the HA.^11^ This might affect host adaptation and viral virulence similar to what has been observed for influenza A.^33^, ^34^ The 26 viruses with full HA sequence data were all collected within the same period (June‐August 2018); therefore, the clustering of the South African Victoria lineage influenza B viruses was based on geographical location (Western Cape vs Gauteng) rather than mutations accumulated over time. The NA analysis followed the HA trend since both are surface proteins under host immune pressure.

The transmissibility of influenza B appeared high for 3 weeks in 2018 compared to historical thresholds (2012‐2017), although influenza severity was moderate throughout most of the season. The high level of the influenza B transmissibility observed in South Africa in 2018 correlates with the observations that double‐deletion influenza B Victoria viruses spread globally within a short period.^11^ It is not known if these viruses could be responsible for more severe influenza seasons in other geographical regions.

Although significant genetic diversity continued to be observed since the emergence of the B Victoria deletion mutants, the two deletion viruses were still well inhibited by antisera raised against the B/Colorado/06/2017‐like viruses (2019 Southern hemisphere vaccine strain).^30^ The B Victoria lineage virus containing three amino acid deletions was poorly inhibited by post‐infection ferret antisera raised against egg‐ and cell culture‐propagated B/Colorado/06/2017‐like viruses, in contrast with viruses with the two amino acid deletions. The viruses with three amino acid deletion are also predominating worldwide. Therefore, the recommended trivalent and quadrivalent 2020 southern hemisphere vaccine strain was changed to include B/Washington/02/2016‐like virus, containing the three amino acid deletions.^31^

No oseltamivir resistance was observed in the South African 2018 influenza B viruses using molecular markers of neuraminidase inhibitor resistance. This finding is not unexpected since neuraminidase resistance or reduced susceptibility is not often detected in influenza B viruses (1%) and has not been detected in South African samples positive for influenza A and B viruses during 2009 to 2013.^25^, ^30^, ^31^ This study only looked at oseltamivir resistance, since oral oseltamivir is available in South Africa, although not routinely used. Resistance should be monitored since it is recommended to treat patients at high risk of developing severe illness.

This study has several limitations that warrant discussion. Firstly, whole‐genome sequencing techniques had low success rates, which could be attributed to failure to sequence viruses with low amplicon concentration (the minimum concentration for input on the MiSeq system was 10 ng/μL).^35^ Secondly, a higher proportion of influenza B lineage inconclusive results were observed in samples from the pneumonia surveillance programme (19%) than the VW programme (9%). This difference was probably due to patients presenting to general practitioners during the acute phase of the infection, resulting in a high viral load of influenza virus in the collected samples. Lastly, although the majority of the viruses were sequenced from the 2018 influenza B season, no influenza B Victoria lineage viruses from September were sequenced. As a result of these limitations, other influenza B Victoria lineages may have circulated in South Africa in 2018, which were not sequenced.

This is the first report of the influenza B Victoria lineage with 162‐163 amino acid deletion mutants in South Africa. This study also suggests that this deletion variant replaced previous circulating influenza B/Victoria viruses. Although this virus appeared more transmissible than influenza B viruses from previous seasons, a severe influenza B season was not seen.

## FUNDING

This research was supported by the National Health Laboratory Service, South Africa, and the United States Centers for Disease Control and Prevention, Atlanta, Georgia, USA under the terms of the co‐operative agreement number: 5U51IP000155.

## CONFLICT OF INTEREST

Cheryl Cohen reports grants from US CDC, grants from Sanofi Pssteur, non‐financial support from Parexel, during the conduct of the study.

Anne von Gottberg, Nicole Wolter, and Sibongile Walaza report grants from US CDC, during the conduct of the study.

## AUTHOR CONTRIBUTIONS

Formal Analysis: Orienka Hellferscee, Meredith McMorrow, Stefano Tempia, Anne von Gottberg

Investigation: Orienka Hellferscee

Methodology: Orienka Hellferscee, Florette Treurnicht, Lucinda Gaelejwe, Alexandra Moerdyk

Project Administration: Gary Reubenson, Cheryl Cohen, Sibongile Walaza

Supervision: Florette Treurnicht, Cheryl Cohen

Visualization: Johanna McAnerney

Writing – Original Draft Preparation: Orienka Hellferscee

Writing – Review & Editing: Orienka Hellferscee, Florette Treurnicht, Lucinda Gaelejwe, Alexandra Moerdyk, Gary Reubenson, Meredith McMorrow, Stefano Tempia, Johanna McAnerney, Sibongile Walaza, Nicole Wolter, Anne von Gottberg, Cheryl Cohen

## TRANSPARENCY STATEMENT

This manuscript is an honest, accurate, and transparent account of the study being reported; that no important aspects of the study have been omitted; and that any discrepancies from the study as planned (and, if relevant, registered) have been explained.”

## Supporting information

**Figure S1.** Influenza B virus detection by lineage per year (A) Viral Watch surveillance and (B) pneumonia surveillance

## Data Availability

Data available on request from the authors.
