# Supplementary material for: Detection of Victoria lineage influenza B viruses with K162 and N163 deletions in the hemagglutinin gene, South Africa, 2018
Source: Health Sci Rep. 2021 Sep 17;4(3):e367. doi: 10.1002/hsr2.367 (PMC8448392; doi:10.1002/hsr2.367)
Supplement: Supplementary file 1 — Figure S1. Influenza B virus detection by lineage per year (A) Viral Watch surveillance and (B) pneumonia surveillance [file HSR2-4-e367-s001.docx]

Supplementary material:

Figure 1: Influenza B virus detection by lineage per year, Viral Watch surveillance (A) and Pneumonia surveillance (B)

A

B
